# Effect of milk-derived bioactive peptides on the lipid stability and functional properties of beef nuggets

**DOI:** 10.1038/s41598-021-04691-w

**Published:** 2022-01-24

**Authors:** Muhammad Sajid Arshad, Gule Hina, Faqir Muhammad Anjum, Hafiz Ansar Rasul Suleria

**Affiliations:** 1 Department of Food Science, Faculty of Life Sciences, Government College University, Faisalabad, Pakistan; 2 University of the Gambia, Serrekunda, The Gambia; 3 School of Agriculture and Food, Faculty of Veterinary and Agricultural Sciences, The University of Melbourne, Parkville, VIC 3052 Australia

**Keywords:** Biochemistry, Lipids, Lipid peroxides

## Abstract

The present study was conducted to ascertain the beneficial effects of bioactive peptides on the oxidative stability and functional properties of beef nuggets. In this study, milk casein protein hydrolysates were extracted and incorporated into beef nuggets which were then subjected to different assessment parameters including oxidative stability, functional capability as well as microbial and physico-chemical quality tests were performed for determining the meat quality at different storage periods. The casein protein hydrolysate powder (CPH) was added at different concentrations in nuggets CPH 2%, 4%, 6% and 8%, with reference to storage period of 0, 5, 10 and 15 days at 4 °C. The results regarding total phenolic contents (TPC) and DPPH free radical scavenging assay showed a significant increased with respect to CPH powder and significantly decreased with respect to storage interval. The TVBN, TBARS and POV of the CPH powder incorporated raw beef nuggets also differed significantly within groups with storage time. Higher POV and TBARS were noticed in the CPH 8% incorporated beef nuggets. However, the raw beef nuggets that were made by the incorporation 8% CPH powder, maintained significantly lower level of TBARS at the end of the storage period in contrast with the levels of the control (CPH 0%). The results of the pH and Hunter color test also showed a significant difference with respect to different groups. The microbiological analysis of beef nuggets showed a significant decrease in the level of both the total aerobic and coliform counts and also indicated a decreasing trend in the level of contamination by these bacteria within the groups. This depicted that the casein protein hydrolysate powder (CPH) or simply, the peptide powder has the strong ability to decrease lipid oxidation and related shelf-life retarding natural processes occurring in the meat. It can also greatly enhance the functional properties of the raw meat (beef) and meat products. Thus, it is seen that the bioactive peptides (BAP’s) are a key factor in improving the oxidative stability and functional properties of beef nuggets.

## Introduction

Bioactive peptides (BAP’s) are known to be as specific organic compounds (proteins) that have a positive impact on body function or conditions that influence on health. Currently, over 1500 totally different BAP’s are reported in an outstanding gen named as ‘Bio-pep’^1^.

Bioactive peptides are organic substances made by amino acids (2–20 amino acids) joined by covalent bonds also known as amide or peptide bonds. Proteins are polypeptides with a greater molecular weight (MW). Bioactive peptides and proteins play vivacious roles in the metabolic functions of living organisms and hence in human health. They exhibit hormone or drug-like activities and can be categorized based on their mode of action as antimicrobial, anti-oxidative, anti-obesity, anti-diabetic, anti-thrombotic, antihypertensive, opioid, immune-modulatory, anticancer, minerals binding and probiotic properties. Consequently, they are released through food processing (fermentation, aging, enzymatic treatment) or during digestion^2^.

Bioactive peptides, commonly known as BAP’s have been appraised as the new generation of biologically active regulators that can halt for example oxidation and microbial degradation taking place in foods. By far, buffalo and bovine milk and dairy products are the significant sources of bioactive proteins and peptides acquired from foods. They can also be derived from other animal sources such as bovine blood^3^, gelatin, meat, eggs and various fish species such as tuna, sardine, herring and salmon. Some vegetal sources of BP and proteins are wheat, maize, soy, rice, mushrooms, pumpkin, sorghum and amaranth^4,5^. Milk proteins possess an ample range of biological activities. For example, immunoglobulins exhibit potent immuno-protective result and lactoferrin (Lf) exhibits medicine activity. Low concentrations of growth factors and hormones, mainly present in colostrum, appear to play an important role in post-natal development^6^.

Most of the functional bioactive peptides are bent in convoluted matrices, consist of variety of hydrolyzed super molecule fractions, their separation and purification are one of the important challenges and are required to study at molecular level. Conventional pressure-driven processes are generally used for amino acids and peptides separation but are restricted by their fouling problems and their low property once separating similar sized biomolecules. In recent times, electro-dialysis using ultra-filtration membranes has been established to fractionate concurrently acidic and basic peptides using a conventional electro-dialysis cell where ion exchange membranes are substituted by ultra-filtration^7^.

Using liquid chromatography–mass spectrometry (HPLC–MS) and tandem mass spectrometry (MS), a large number of medium and low-MW BAP’s (including opioid and phosphopeptides) were recognized in human milk from mothers of pre and full-term infants. The development of many peptides ratifies the larger susceptibility of human milk to casein proteolysis compared to bovine milk. Fermentation of milk proteins mistreatment carboxylic acid bacterium (LAB) is an appealing approach to come up with purposeful foods supplemented with BP given the low price and positive organic process image allied with fermented milk products^8^.

However, some discrepancies on the nature of BAP’s depending on the milk protein source, the isolation, and categorization of peptides of different bioactivities from milk protein hydrolysates and products of buffalo, camel, goat, mare, sheep, and yak milk have already been informed^9^. Bioactive peptides having high antioxidant activity are gained from fermented food products through the action of different beneficial micro-organisms and endogenous proteolytic enzymes^10,11^.

Bioactive peptides are claimed to prevent lipid oxidation in meat and other products; in addition, these antioxidant peptides have also been reported to possess substantial health-promoting potential besides controlling oxidation. Peptides derived from milk proteins have shown antioxidant properties that prevent the per-oxidation of essential fatty acids. Whey as well as casein protein hydrolysates have been shown to inhibit lipid oxidation in muscle foods^12^.

The antioxidative potential of peptides can be accredited to their metal ion-chelation, lipid per-oxidation inhibition, and radical-scavenging properties. It also has been documented that the structure of peptides with its amino acid sequence can influence their anti-oxidative potential^13^. Thus, peptides and proteins have tremendous potential as antioxidants for food, especially in meat and meat products^14^. Keeping in view the above features the objectives set to be attained for present research includes the extraction of milk peptides and their incorporation in meat and assessing the effect of these bioactive peptides on the functional parameters of meat and evaluating the oxidative stability of meat in response to the addition of bioactive peptides.

## Materials and methods

### Procurement of raw materials

Beef was sourced from the superstore (METRO, Pakistan) and this meat was then ground finely for further use. The ground beef samples were then stored in an airtight bag for further study. The milk-protein isolates (MPIs) had a dry basis purity of 90%. The different standards and reagents used were purchased from renowned companies like Sigma-Aldrich (Tokyo, Japan) and Merck (Merck KGaA and Darmstadt, Germany).

### Preparation of milk protein hydrolysates

According to the peptide guidelines of^15^, three different types of microbial proteases namely the effective enzyme Validase (Val), Alkaline Protease (AP) and Neutral Protease (NP) were used to hydrolyze the milk protein isolates. Thus, an estimated amount of 50 g of milk protein was homogenized in 500 mL of water and it was hydrolyzed by each protease at a concentration of about 2% (w/w, enzyme/substrate). The enzymatic hydrolysis was carried out in a water bath for 6 h, and the mixture was shaken at 55 °C. The mixture was centrifuged at 15,000*g* and the remaining soluble fraction was filtered using Whatman filter paper for further classification.

### Separation of protein hydrolysates by ultra-filtration

The casein hydrolysate prior to dilution with distilled water was ultra-filtered using the protocol of^15^ for a nominal molecular weight limit (NMWL) of 10 kD membrane under a pressure of 40 psi, utilizing nitrogen. As a result of this process, two fractions retentate and permeate were obtained. The entire retentate and permeate were lyophilized and stored at − 20 °C for further analysis.

### Preparation of CPH powder

For the preparation of buffalo milk casein protein hydrolysate (CPH) powder, the method described earlier by Kumar et al.^16^ was made in use. In short, the casein protein hydrolysate (CPH) powder containing almost 5% of the total solids and which was already freeze-dried, was made to dissolve for the highest optimum enzymatic action within the phosphate buffer having a pH 8.0 and then about 1:100 (Enzyme: Substrate ratio) of alkaline protease was added to it. Afterwards, by incubating the sample at an unceasing temperature of 55 °C, the process of hydrolysis was conducted in a stirred water-bath for a period of 6 h. The sample obtained after the completion of hydrolysis was then heated rapidly to about 85 °C in a water-bath aimed at 15 min, in order to denature the residual enzyme in it. After this, the sample was cooled down and instantaneously centrifuged at 11,200×*g* in a refrigerated centrifuge machine for 25 min and the subsequent supernatant was then collected and stored. It was then dried in a vacuum oven at 600 mmHg vacuum and then oven was run at 50 °C for the duration of about 12–14 h. The supernatant was ground in the form of a fine powder and it was kept at − 20 °C till its next use.

### Formulation of meat nuggets

The peptide powder thus formed was added in meat that was then made into nugget each weighing 16 g at different concentrations. The formed nuggets were then packed in zipper bags and stored at refrigeration temperature following the protocols of^17^. Composition of peptide powder incorporate raw beef nuggets: five treatment groups were made and each group contained the casein protein hydrolysate (CPH) powder in quantities as CPH 0%, made without the incorporation of peptide powder regarded as the control sample, CPH 2% containing 2% peptide powder, CPH 4% containing 4% peptide powder, CPH 6% and CPH 8% containing 6% and 8% protein peptide powder respectively. The treatments were then subjected to various functional capability and oxidative stability tests at 0, 5, 10 and 15 days interval.

### Total phenolic contents (TPCs)

The TPC content in the beef nuggets was calculated by using the Folin-Ciocalteu method as defined in our previous published method of Suleria et al.^18^. 25 µL extract and same amount of Folin-Ciocalteu reagent solution (1:3 diluted with water) respectively and 200 µL of water added in plate having 96 well. The prepared mixture was shifted for the incubation for 5 min in the dark room at controlled temperature (~ 25 °C). 25 µL of 10% (w:w) sodium carbonate was added into the reaction mixture and then shifted for incubation in dark room for 1 h at 25 °C. Spectrometer used for absorbance and measured at 765 nm. Gallic acid standard in concentrate form having range of 0–200 µg/mL was used to prepare the standard curve, gallic acid TPC content was expressed in mg equivalents/g on the basic of fresh weight (f.w.) (mg GAE/g of sample).

### Antioxidant capability using DPPH

The beef patties samples made with CPH powder were subjected to analysis of the DPPH radical scavenging activity according to the procedure outlined by Brand-Williams et al.^19^. The antioxidant activity of the homogenized meat samples were assessed by measuring their scavenging abilities to DPPH stable radicals. A sample of 125 μL was mixed with 0.0012 m DPPH solution followed by the addition of 95% MeOH up to a final volume of 4 mL and allowed to rest for 1 h at room temperature. The absorbance of the resulting solution and the blank were recorded spectrophotometrically at 515 nm. The inhibition of free radicals by DPPH in percent (%) was calculated by using the following formula.

$${\text{Inhibition }}\left( \% \right) \, = 100 \times ({\text{Absorbance blank}} - {\text{ Absorbance sample}}/{\text{Absorbance blank}})$$

### Total volatile basic nitrogen (TVBN)

The TVBN value of beef nuggets was measured by following the method of An et al.^20^ with some modification. Homogenization of 5 g meat sample was done in 45 ml distilled water for 30 s and 5 ml of the prepared homogenate was used to mix with an equal volume of 10% trichloroacetic acid (TCA; w/v in distilled water). For the determination of TVBN Value prepared TCA were used. 1 ml of TVBN reagent used for inner well and the same amount used for outer well of the Conway unit by following the usage of 1 ml saturated potassium carbonate (K_2_CO_3_). After that, Conway unit was closed using airtight ground glass plate, and then shifted for the incubation at controlled temperature for 3 h. TVBN reagent was again titrated using 0.02 N (H_2_SO_4_) till blue color converted into pink. Following equation was used for the calculation of TBVN value.

$${\text{``N'' mg}}/{\text{ml of extract}} = \, 14 \, \times {\text{a }} \times {\text{ b}}$$

$${\text{TVBN value}}\left( {{\text{mg}}/100\;{\text{ml}}} \right) \, = \, 100 \, \times {\text{ N}}$$

14 = molecular weight of nitrogen, a = normality of H_2_SO_4,_ b = volume of H_2_SO_4,_ (titration value).

### Peroxide value (POV)

Peroxide value was determined according to the method of the International Dairy Federation (IDF)^21^. The iron (II) chloride solution was prepared by using 0.4 g barium chloride dihydrate dissolved in 50 mL water. This solution was added slowly and with constant stirring to an iron (II) sulfate solution (0.5 g FeSO_4_·7H_2_O dissolved in 50 mL water). (1) 2 mL of 10 N HCl was added to the resulting solution. (2) The barium sulfate precipitate was filtered off to give a clear iron (II) solution, which was stored in a brown bottle and kept in the dark. The ammonium thiocyanate solution was prepared as under: 30 g ammonium thiocyanate was dissolved in water, and the volume was made up to 100 mL. The samples (0.2 g) was homogenized with 9.8 mL of cold phosphate buffer (40 mM, pH 6.8) for 10 s at 13,000 rpm, then mixed in a disposable glass tube with 9.8 mL chloroform–methanol (7:3, v/v) on a vortex mixer for 2–4 s, then added ammonium thiocyanate solution (50 μL) and the sample was vortex-mixed for 2–4 s, then added 50 μL iron (II) solution and the sample was again vortex-mixed for 2–4 s, After 5 min incubation at room temperature, the absorbance of the sample was determined at 500 nm against a blank that contained all the reagents except the sample by using a spectrophotometer.

The entire procedure was conducted in subdued light and completed within 10 min.

$$Peroxide\;value = \frac{{\left( {{\text{A}}_{{\text{s}}} - {\text{A}}_{{\text{b}}} } \right) \times {\text{m}}}}{{55.84 \times {\text{m}}_{0} \times 2}}$$

A_s_ = Absorbance of the sample, A_b_ = Absorbance of the blank, M (Standard) = 41.52, m_0_ = mass in gram, 55.84 = atomic weight of iron.

### Thio-barbituric acid derivative quality test (TBARS)

The lipid oxidation in beef patties was measured by using a method of TBARS as described by Ahn et al.^22^. Fifteen milliliters of deionised distilled water (DDW) was added to the beef patties samples (5 g), and homogenized for 30 s. The homogenate (1 mL) was transferred to a disposable test tube and 50 μL of butylated hydroxytoluene (BHT) and 2 mL of 2-thiobarbituric acid and trichloracetic acid mixture (20 mL 20 mM TBA in 15% trichloroacetic acid) solution were added. After that, the mixture was vortexed and incubated in a boiling water bath for 15 min, then the sample was cooled in cold water for 10 min and after that the sample was centrifuged for 15 min at 2500×*g* at 4 °C. Then check the absorbance of the resulting supernatant was measured using a UV/VIS spectrophotometer at 532 nm against a blank containing 1 mL DW and 2 mL TBA/TCA solution.

The concentration of malonaldehyde was calculated from the following equation. TBARS values were expressed as mg malondialdehyde per kg meat.

$${\text{mg malondialdehydes per kg}}\;{\text{meat}} = \frac{{\left( {{\text{Sample}}\;{\text{absorbance }} - {\text{ blank}}} \right) \times {\text{ Total sample vol}}}}{0.000156 \times 1000}.$$

### Physico-chemical analysis (pH and color)

The pH values of beef nuggets were ascertained with a digital pH meter by way of the procedure devised by^23^. The color of beef nuggets was tested by using the Hunter color (Chromameter, CHROMA-400, Pulsed xenon lamp, 400 nm to 700 nm, Japan) as method of Holmon et al.^24^. The sensor has 14.0 mm aperture and 45° measuring geometry. Illuminant D65 and 10° standard observer settings were used. The CIE L* (light-ness), a* (redness) and b* (yellowness) values were obtained directly without removing the coating by directly attaching the lens on the surface of the sample. 3 pieces of beef nuggets were measured for each treatment and measured at different locations and this sense 9 data were collected.

### Microbial quality determining assays

The microbial counts of the total aerobic bacteria (TAB) and coliforms of the stored beef nuggets were calculated using the methods in accordance with the guidelines of Association of Official Agricultural Chemists^25^. 10 g of meat sample was homogenized with 0.1% of sterile peptone water. Serial dilutions were prepared in 0.1% peptone water and plated with plate count agar which were incubated at 37 °C for 48 h. The colonies were counted and expressed as log CFU/g.

### Statistical analysis

Statistical analysis was performed on the collected data in order to determine the level of significance. The data of different parameters were represented as the means ± standard deviation and two-way analysis of variance (ANOVA) was used to test for differences in mean values between different samples, followed by Tukey’s honestly significant differences (HSD) multiple rank test at *p* < 0.05. ANOVA was performed by Minitab Program for Windows version 18.0 elaborated by^26^ (Minitab, LLC, State College, PA, USA).

## Results and discussion

### Total phenolic contents (TPC)

The incorporation of casein protein hydrolysate (CPH) powder in raw beef nuggets depicted a significant increased (p < 0.05) in total phenolic contents of the beef nuggets (Table 1). The results showed that higher TPC value was observed in the group that was incorporated with 8% CPH powder, at the beginning of the storage period while the lowest value was shown by the control. The total phenolic contents increased significantly (p < 0.05) within groups, with the increase in CPH powder and advancement in storage time except for the control group at day 15. At the end of the storage period, the highest TPC were observed in group having 8% CPH powder whereas the lowest amount of TPC was shown by the control at the end of the storage period. It is thus considered that during the course of refrigeration storage (4 ± 1 °C), the total phenolic contents decreased significantly in all the treatment groups during the storage period but the casein protein hydrolysate powder (CPH) incorporated treatment groups showed a slower rate of decrease than the control, which indicates that the CPH powder incorporated beef nuggets possess higher oxidative stability than the control. These results are in agreement with the findings of^27^ who reported that a significant decrease in the total phenolic contents of all the fresh chicken sausages and sausage products, at each interval of the storage period.

**Table 1:** Total phenolic content of casein protein hydrolysate (CPH) incorporated beef nuggets during storage.

Groups	TPC (mg GAE/g)			
	Storage days			
	0	5	10	15
Control	95.66 ± 2.41eB	102.00 ± 2.00eA	101.50 ± 1.91eA	92.00 ± 1.99eC
CPH2%	110.00 ± 2.22 dB	114.50 ± 2.19dA	111.00 ± 2.30 dB	109.00 ± 1.69dC
CPH4%	118.50 ± 2.11cB	122.50 ± 2.20cA	117.50 ± 1.35cC	114.50 ± 1.80cD
CPH 6%	122.00 ± 2.10bB	128.00 ± 2.18bA	123.50 ± 1.30bB	120.00 ± 2.66bC
CPH8%	126.00 ± 2.00aC	130.50 ± 2.15aA	127.00 ± 1.35aB	124.50 ± 2.65aD

CPH: casein protein hydrolysate. All the values are means of triplicate determinations ± Standard Deviation (SD). Means within the column with different small letters are significantly different (p < 0.05) and different capital letters within the row differed significantly.

### DPPH free radical scavenging assay

In this study, the DPPH scavenging assay was conducted as the first and foremost measure to determine the anti-oxidant activity of casein protein hydrolysates (CPH) powder present in beef nuggets. The incorporation of casein protein hydrolysate (CPH) powder in the raw beef nuggets caused a significant increased (p < 0.05) in the free-radical scavenging activity (DPPH) of the beef nuggets (Table 2). The results showed that the highest DPPH value was observed in the group that was incorporated with 8% CPH powder at the beginning of the storage period while the lowest value was shown by the control. The DPPH free radical scavenging activity increased significantly (p < 0.05) within groups, in all the samples with the increase in storage time. At the end of the storage period, the highest DPPH value was observed in the treatment group having 8% CPH powder whilst the lowest DPPH value was shown by the control at the end of the storage period. The results of the present study are in accordance with the findings of^28^, who also reported significantly higher DPPH radical scavenging activity of the pork sausages enriched with synthetic anti-oxidants and mechanically deboned chicken meat hydrolysates. Similar results were also reported by^29^ for the flesh hydrolysates of tannery. In addition^30,31^ also showed by their results that the casein calcium peptides (CCP’s) are free-radical scavengers, especially of the peroxyl radical which is the chief propagator of the oxidation chain reaction of fats, thus ending the chain reaction. The anti-oxidant activity of meat has also been related to the casein-derived peptides the human milk^32^.

**Table 2:** DPPH of casein protein hydrolysate (CPH) powder incorporated beef nuggets during storage.

Groups	DPPH (%)			
	Storage days			
	0	5	10	15
Control	66.71 ± 0.38eA	62.14 ± 0.30eB	58.41 ± 1.28eC	52.13 ± 0.56eD
CPH 2%	71.16 ± 0.28dA	65.19 ± 0.18 dB	61.51 ± 1.31dC	57.71 ± 0.43dD
CPH 4%	76.74 ± 0.31cA	69.99 ± 1.50cB	64.09 ± 1.16cC	60.09 ± 0.23cD
CPH 6%	81.61 ± 0.28bA	73.32 ± 1.21bB	69.19 ± 1.23bC	65.16 ± 0.17bD
CPH 8%	85.98 ± 0.44aA	79.18 ± 0.50aB	75.34 ± 0.51aC	70.01 ± 0.33aD

CPH: casein protein hydrolysate. All the values are means of triplicate determinations ± Standard Deviation (SD). Means within the column with different small letters are significantly different (p < 0.05) and different capital letters within the row differed significantly.

### Total volatile basic nitrogen (TVBN)

The TVBN of beef nuggets incorporated with CPH powder showed significant variation with respect to treatment and storage intervals. The results thus acquired showed that the highest value of TVBN was observed in the control group (CPH 0%) at the beginning of the storage period while the lowest value was shown by CPH 8% (Table 3). The TVBN value decreased significantly (p < 0.05) within groups, in all the samples with the increase in storage time. At the end of storage period (at day 15), the highest TVBN value was observed in the beef nuggets that were made without the incorporation of casein protein hydrolysate (CPH) powder which was the control group (CPH 0%), followed by CPH 2% whilst the lowest TVBN value was observed in the group incorporated with 8% CPH powder, at the end of the storage period. The results of present study are in agreement with the findings of^33^, who stated that after storage intervals, the TVBN contents of the additives containing soybean protein hydrolysate were lower as compared to the control samples but were not significantly different. Similar observations were reported by Al. Bachir and Mehiob, who found the same effect of TVBN with the irradiated buffalo meat. Abu Saleem and Banaru^34^ also demonstrated that soy protein coating effectively retarded lipid oxidation in pre-cooked beef patties.

**Table 3:** TVBN of casein protein hydrolysate (CPH) incorporated beef nuggets during storage.

Groups	TVBN (mg/1000 g)			
	Storage days			
	0	5	10	15
Control	10.37 ± 1.53aD	18.45 ± 0.15aC	26.05 ± 1.02aB	30.05 ± 2.50aA
CPH2%	7.87 ± 2.07bD	16.53 ± 0.33bC	22.64 ± 0.41bB	27.05 ± 1.04bA
CPH4%	6.37 ± 2.00cD	12.33 ± 0.77cC	20.87 ± 1.22cB	24.45 ± 0.25cA
CPH6%	5.12 ± 2.06dD	11.53 ± 0.82dC	16.65 ± 0.35 dB	21.99 ± 0.33dA
CPH8%	5.00 ± 2.08dD	10.47 ± 0.71eC	14.25 ± 0.51eB	19.26 ± 0.28eA

CPH: casein protein hydrolysate. All the values are means of triplicate determinations ± Standard Deviation (SD). Means within the column with different small letters are significantly different (p < 0.05) and different capital letters within the row differed significantly.

### Peroxide value (POV)

Peroxide value of beef nuggets with CPH powder depicted significant difference with respect to treatment and storage. The results showed that the highest value of per-oxidation was observed in the control group at the beginning of the storage period while the lowest value was shown by CPH 8% (Table 4). The POV increased significantly (p < 0.05) in all the samples with the increase in storage time. At the end of the storage period (day 15), the highest POV was observed in the beef nuggets that were made without the incorporation of casein protein hydrolysate (CPH) powder i.e. control whilst the lowest POV was shown by CPH 8% which significantly indicates the strong anti-oxidant and anti-microbial potential of casein protein hydrolysates, in plummeting the lipid oxidation which is evident from the previous experimental reports of^16^. The results of our study are in agreement with the findings of^35^ who reported that decreased in the peroxide value (POV) in the grass carp mince during storage, with the incorporation of grass carp protein hydrolysates and cracklings hydrolysates in the pork meat balls as reported by^36^. The significantly strong suppression of the lipid oxidation reaction, in both POV and TBARS, by the incorporation of hydrolysed potato protein in the cooked beef patties was reported in another study conducted by^12,37^.

**Table 4:** POV of casein protein hydrolysate (CPH) incorporated beef nuggets during storage.

Groups	POV (meq peroxides/kg)			
	Storage days			
	0	5	10	15
Control	0.64 ± 0.05aD	0.68 ± 0.08aC	0.74 ± 0.02aB	0.80 ± 0.04aA
CPH 2%	0.62 ± 0.04bD	0.65 ± 0.05bC	0.72 ± 0.12bB	0.78 ± 0.03bA
CPH 4%	0.58 ± 0.02cD	0.65 ± 0.04bC	0.73 ± 0.22bB	0.76 ± 0.03cA
CPH 6%	0.54 ± 0.06dD	0.63 ± 0.03cC	0.70 ± 0.15cB	0.74 ± 0.02dA
CPH 8%	0.55 ± 0.03dD	0.60 ± 0.02dC	0.68 ± 0.06 dB	0.70 ± 0.01eA

CPH: casein protein hydrolysate. All the values are means of triplicate determinations ± Standard Deviation (SD). Means within the column with different small letters are significantly different (p < 0.05) and different capital letters within the row differed significantly.

### Thio-barbituric acid reactive substances (TBARS)

The TBARS value of beef nuggets depicted significant variation between treatments and storage intervals. However, the raw beef nuggets that were made by the incorporation 8% casein protein hydrolysate (CPH) powder, maintained significantly lower level of TBARS at the end of storage in contrast with the levels of the control (CPH 0%). The results thus obtained depicted highest TBARS value in control sample at the start of the storage period while the lowest value was shown by the beef nuggets that were incorporated with 8% casein protein hydrolysate powder (Table 5). The TBARS value increased significantly (p < 0.05) in all the samples during storage. At the end of storage period, the highest value of TBARS was observed in the nuggets that were not incorporated with the peptide powder i.e. the control group (CPH 0%) with the value 0.53 ± 0.04 MDA/kg, followed by CPH 2% while the lowest value of the TBARS assay was shown by CPH 8%, at the end of the storage period. The maintenance of lower TBARS values in the CPH powder incorporated treatments might be due to the presence of potent anti-oxidant activity of the casein protein hydrolysates. The results obtained are in accordance with the previous experimental reports of^38^. The results of the present research are also supported by the results of^16^ in the ground beef and mechanically deboned poultry meat incorporation with casein protein hydrolysate (CPH). Abu Saleem and Banaru^34^ also demonstrated that soy protein coating effectively retarded lipid oxidation in pre-cooked beef patties. Earlier research also reported that MDA concentrations higher than 0.5 mg/kg are the threshold value for the perception of rancidity by consumers^39^. These results are also in agreement with the results reported by^40^, for other anti-oxidants applied to the meatballs.

**Table 5:** TBARS of casein protein hydrolysate (CPH) powder incorporated beef nuggets during storage.

Groups	TBARS (MDA/kg)			
	Storage days			
	0	5	10	15
Control	0.43 ± 0.03aD	0.46 ± 0.04aC	0.50 ± 0.05aB	0.53 ± 0.04aA
CPH2%	0.39 ± 0.04bD	0.42 ± 0.03bC	0.46 ± 0.04bB	0.49 ± 0.05bA
CPH4%	0.38 ± 0.04bD	0.42 ± 0.03bC	0.43 ± 0.02cB	0.45 ± 0.03cA
CPH6%	0.34 ± 0.03cD	0.41 ± 0.03cC	0.43 ± 0.02cB	0.46 ± 0.03cA
CPH8%	0.30 ± 0.01dD	0.36 ± 0.02dC	0.39 ± 0.03 dB	0.43 ± 0.02dA

CPH: casein protein hydrolysate. All the values are means of triplicate determinations ± Standard Deviation (SD). Means within the column with different small letters are significantly different (p < 0.05) and different capital letters within the row differed significantly.

### Physico-chemical tests

#### pH

The pH of the raw beef nuggets incorporated with different concentrations of casein protein hydrolysate (CPH) powder didn’t vary significantly, at the beginning of the storage period (on day 0) but it varied significantly (p < 0.05) on the 5th day of storage (Table 6). The results thus obtained showed that the highest pH value was recorded for the treatment group that was incorporated with 8% casein protein hydrolysate (CPH) powder, at the beginning of the storage period followed by CPH 6% while the lowest pH value was shown by the control group (CPH 0%). The value of pH increased significantly (p < 0.05) within groups except for CPH 8%, with advancement in storage duration but this increase in the pH value of CPH powder incorporated beef nuggets was significantly lower than the control group. At the end of the storage period, the highest pH was observed in the control group, followed by CPH 2% whereas the lowest value of pH was observed in the group having 8% CPH powder.

**Table 6:** pH of casein protein hydrolysate (CPH) powder incorporated raw beef nuggets.

Groups	pH			
	Storage days			
	0	5	10	15
Control	5.70 ± 0.07dD	6.23 ± 0.08aC	6.41 ± 0.02aB	6.71 ± 0.05aA
CPH2%	5.81 ± 0.03cD	6.15 ± 0.17bC	6.24 ± 0.21bB	6.54 ± 0.01bA
CPH4%	5.79 ± 0.04cD	6.01 ± 0.20cC	6.13 ± 0.32cB	6.23 ± 0.12cA
CPH6%	5.84 ± 0.08bC	5.85 ± 0.16dC	5.97 ± 0.16 dB	6.09 ± 0.19dA
CPH8%	5.91 ± 0.06aB	5.71 ± 0.11eD	5.81 ± 0.12eC	5.94 ± 0.19eA

CPH: casein protein hydrolysate. All the values are means of triplicate determinations ± Standard Deviation (SD). Means within the column with different small letters are significantly different (p < 0.05) and different capital letters within the row differed significantly.

The pH of casein protein hydrolysate (CPH) powder incorporated treatment groups as well as control followed an increasing trend throughout the storage period except for CPH 8% on day 5, but the rate of the increase in pH of casein protein hydrolysate (CPH) powder incorporated treatments was lower than that of the control group. Thus, the pH recorded in the present study is completely in the range as reported by^41^ in ground chevon (lamb meat) during the refrigerated storage (4 ± 1 °C). Sharma et al.^28^ also stated the increase in pH of pork sausages that were incorporated with mechanically deboned chicken meat hydrolysates (MDCM). They also reported a dose-dependent, lower rate of pH increment in the MDCM hydrolysate incorporated products.

#### Color

Color is one of the principal and strong physical parameter for determining the extent of spoilage in the meat. The incorporation of the peptide powder at different concentrations may influence the color profile due to its contact with the other food components and with the passage of time it also changes because of lipid oxidation and microbial growth in the meat and meat products. Hence, the instrumental color profile analysis turns out to be an important entity for studying the change in color profile during the period of storage. Objective meat color is generally measured in the CIE L*a*b* color space^24,42^.

The results thus obtained showed that the value of lightness decreased significantly (p < 0.05) among groups along the storage duration. The higher value of lightness was observed at the beginning of the storage period (at day 0) in the beef nuggets that were incorporated with 8% casein protein hydrolysate (CPH) powder while slightly lower value was shown by the control group (CPH 0%) (Table 7). The value of lightness decreased significantly in all the samples with the increase in storage time. In contrast to lightness, the redness or a* value increased significantly (p < 0.05) in all the treatment groups, the higher value of redness was observed in the control group (CPH 0%) while lower value was observed in the group containing 8% casein protein hydrolysate powder. As far as the yellowness or b* value is concerned, a significant decrease was observed in the yellowness of control with the increase in the storage time. Higher value of yellowness was shown by the control group (CPH 0%) whereas a lower value was observed in the group that was incorporated with 8% casein protein hydrolysate (CPH) powder at the start of storage period (at day 0). At the end of the storage period, the highest value of lightness was observed in the beef nuggets made without the incorporation of CPH powder whilst the lowest value of lightness was shown by CPH 8%, in contrast to lightness the redness followed a decreasing trend at the end of storage period (at day 15), the highest a* value was shown by CPH 8% whereas the lowest value was shown by the control group (CPH 0%). Similarly, the yellowness or b* value of casein protein hydrolysate (CPH) powder incorporated beef nuggets followed a decreasing trend. At the end of the storage period (at day 15), the highest b* value was observed in the group that was incorporated with 8% casein protein hydrolysate (CPH) powder whilst the lowest b* value was shown by the control group (CPH 0%). This indicated that even though, the CPH powder incorporated beef nuggets had to some extent, lower b* value on the initial days but it was retained during the course of time and this might be due to the added anti-oxidant properties of casein protein hydrolysate powder. The results of the present study are in accordance with the findings of^43^ who also reported that the meat emulsions that were incorporated with hydrolyzed potato protein (HPP) were darker (i.e. lower value of L*) as compared to those made without the incorporation of HPP. Similar results, where the yellowness (b*) of meat emulsions incorporated with hydrolyzed potato protein had lower values than those made without HPP were reported as stated earlier by^43^ are in agreement with the results of our study.

**Table 7:** Color of casein protein hydrolysate (CPH) powder incorporated raw beef nuggets.

Groups	Color			
	Storage days			
	0	5	10	15
	L* (lightness)			
Control	50.00 ± 0.99dA	48.03 ± 1.15cB	46.05 ± 1.46aC	44.29 ± 1.09aD
CPH 2%	50.09 ± 1.39dA	48.00 ± 0.98cB	45.73 ± 1.05cC	43.90 ± 1.75bD
CPH 4%	50.17 ± 1.37cA	48.02 ± 1.11cB	45.83 ± 0.91bC	43.79 ± 1.60bD
CPH 6%	50.37 ± 1.43bA	48.24 ± 1.17bB	45.86 ± 0.92bC	42.54 ± 1.91cD
CPH 8%	50.42 ± 1.52aA	48.35 ± 1.95aB	45.89 ± 1.18bC	42.24 ± 1.49dD
	a* (redness)			
Control	7.44 ± 0.45cA	7.22 ± 0.31 dB	6.17 ± 0.61eC	5.55 ± 0.15eD
CPH 2%	7.40 ± 0.22dA	7.35 ± 0.77cA	6.26 ± 0.32 dB	5.60 ± 0.35dC
CPH 4%	7.53 ± 0.33bA	7.36 ± 0.77cB	6.35 ± 0.41cC	5.65 ± 0.34cD
CPH 6%	7.56 ± 0.36bA	7.42 ± 0.11bB	6.45 ± 0.22bC	5.75 ± 0.55bD
CPH 8%	7.60 ± 0.38aA	7.51 ± 0.33aB	6.65 ± 0.30aC	5.80 ± 0.50aD
	b* (yellowness)			
Control	12.99 ± 0.52aA	12.00 ± 0.15dA	10.18 ± 0.10cB	9.60 ± 0.19dC
CPH 2%	12.97 ± 0.55aA	12.05 ± 0.16cB	11.17 ± 0.11cC	10.06 ± 0.39cD
CPH 4%	12.92 ± 0.43bA	12.09 ± 0.17bB	11.26 ± 0.20bC	10.17 ± 0.64bD
CPH 6%	12.64 ± 0.61cA	12.11 ± 0.55bB	11.31 ± 0.23bC	10.25 ± 0.55aD
CPH 8%	12.62 ± 0.60cA	12.17 ± 0.52aB	11.41 ± 0.30aC	10.35 ± 0.45aD

CPH: casein protein hydrolysate. All the values are means of triplicate determinations ± Standard Deviation (SD). Means within the column with different small letters are significantly different (p < 0.05) and different capital letters within the row differed significantly.

In case of redness (a* value), the results of our research were in accordance with the results of^44^ who also reported loss of redness values in the red muscle due to oxidation.

### Microbial analysis

#### Total coliforms count (TCC)

The total coliform count analysis was carried out in order to determine the microbial count of pathogenic flora. The results acquired showed that the highest coliform count was found in the control group (CPH 0%), at the beginning of the storage period while the lowest coliform count was found in the treatment group that was incorporated with 8% of casein protein hydrolysate (CPH) powder (Table 8). The total coliforms count decreased significantly (p < 0.05) within groups up to day 5 except the control group but this decrease in the coliform counts of the CPH powder incorporated beef nuggets was significantly higher at day 5. At the end of the storage period, the highest coliform count was observed in the control group, followed by CPH 2% whereas the lowest coliform count was found in the treatment group incorporated with 8% CPH powder.

**Table 8:** Total coliforms count (TCC) of casein protein hydrolysate (CPH) powder incorporated raw beef nuggets.

Groups	Total coliforms count (log10 CFU/g)			
	Storage days			
	0	5	10	15
Control	8.01 ± 1.00aD	8.68 ± 2.30aC	9.17 ± 0.50aB	10.27 ± 2.09aA
CPH 2%	7.88 ± 1.28bD	8.00 ± 1.99bC	8.67 ± 1.04bB	9.12 ± 2.10bA
CPH 4%	7.44 ± 1.45cD	7.94 ± 1.50cC	8.47 ± 1.25cB	8.89 ± 2.07cA
CPH 6%	6.65 ± 1.12dD	6.90 ± 1.09dC	7.77 ± 2.03 dB	8.17 ± 2.35dA
CPH 8%	5.59 ± 1.25eD	6.29 ± 1.29eC	7.27 ± 1.47eB	7.97 ± 2.50eA

CPH: casein protein hydrolysate. All the values are means of triplicate determinations ± Standard Deviation (SD). Means within the column with different small letters are significantly different (p < 0.05) and different capital letters within the row differed significantly.

It became clear from the results of the present study that the total coliform counts (TCC) decreased significantly (p < 0.05) at day 5 in all the treatment groups incorporated with CPH powder but the difference was more obvious in the last two treatment groups (CPH 8% and CPH 6%), having higher concentration of CPH powder. After that, a significant increase in the total coliform count was seen in all the treatment groups as well as the control group. However, the casein protein hydrolysate (CPH) powder incorporated treatment groups showed significantly lower counts of coliforms, at the end of the storage period. The results of the present study are comparable with the results reported by^45^ in hot deboned pork incorporated with lactoferrin and casein peptides. Umuhumuza et al.^46^ also studied the supplementation of various hydrolyzed proteins isolated from skimmed milk and their effects on the growth of probiotic bacteria and established that the fraction and type of protein which is used directly, affects the rate and growth of micro-organisms.

#### Total aerobic bacteria (TAB)

The microbial counts, in the casein protein hydrolysate (CPH) powder incorporated treatment groups and control group were analyzed for the estimation of Total Aerobic Bacteria (TAB). The results showed that the highest TAB count was found in control at the beginning of the storage period while the lowest TAB count was found in the treatment group that was incorporated with 8% casein protein hydrolysate (CPH) powder (Table 9). The TAB count increased significantly (p < 0.05) within groups until day 15, except on day 0 but this increase in the Total Aerobic Bacteria counts of the CPH powder incorporated beef nuggets was significantly lower throughout the storage period. At the end of the storage period, the highest TAB count was observed in the control group (CPH 0%), followed by CPH 2% whilst the lowest TAB count was found in the treatment group that was incorporated with 8% CPH powder. The results thus obtained indicated an elevated count of the TAB and in the control group (CPH 0%) on the 15th day of refrigeration storage while a lower TAB count was observed in the group that was incorporated with CPH 8%.

**Table 9:** Total aerobic bacteria (TAB) of casein protein hydrolysate (CPH) powder incorporated raw beef nuggets.

Groups	Total aerobic bacteria (log 10 CFU/g)			
	Storage days			
	0	5	10	15
Control	8.35 ± 0.10aD	9.24 ± 0.30aC	10.22 ± 0.02aB	10.94 ± 0.24aA
CPH 2%	6.89 ± 0.29bD	7.37 ± 0.47bC	8.36 ± 0.22bB	8.96 ± 0.36bA
CPH 4%	5.50 ± 0.32cD	6.37 ± 0.37cC	6.94 ± 0.12cB	7.27 ± 0.28cA
CPH 6%	4.25 ± 0.20dD	5.42 ± 0.24dC	6.37 ± 0.19cB	7.17 ± 0.22cA
CPH 8%	3.30 ± 0.30eD	4.83 ± 0.28eC	5.25 ± 0.36 dB	6.35 ± 0.19dA

CPH: casein protein hydrolysate. All the values are means of triplicate determinations ± Standard Deviation (SD). Means within the column with different small letters are significantly different (p < 0.05) and different capital letters within the row differed significantly.

It became clear from the results of our study that with the increase in concentration of the CPH powder, the microbial population of the aerobic bacteria decreased significantly in groups and these results also showed that a higher concentration of CPH powder incorporated in beef nuggets, retards the growth of the Total Aerobic Bacteria (TAB). The results of our study are in accordance with the results of^47^, which indicated that the higher anti-oxidant and anti-microbial activity of the whole hydolysates may be due to the presence of the peptides of different sizes and charges which contribute to the overall activity of the protein hydrolysates.

#### Correlation and stepwise regression study

The correlation and stepwise regression study for beef nuggets is shown in Tables 10 and 11. Correlation study showed that Total phenolic content was significant positive associated with DPPH activity of beef nuggets and negatively associated with TBARS value, total coliform and total bacterial count. However, TVBN, POV pH and color values were found non-significant correlation. However, DPPH activity of CPH treated meat had highly significant negative association with TVBN, POV, TBARS, pH, Coliform and Total Bacterial Counts whereas, color value of stored meat showed positive association with DPPH activity. TVBN value showed the highly positive correlation with POV, TBARS, pH, Coliform, TBC of treated meat with CPH powder whereas, color value showed negative association with TVBN value. Similarly, POV value significantly increased as the TBARS, pH of meat was increased with the storage along with bacteria load (total coliform and total bacterial count). Whereas, color value showed deterioration with the increased of POV values. Likewise, TBARS value of CPH treated meat during storage showed the positive association with pH value of meat, bacterial load (both coliform and total bacterial count). As the pH value of the meat increased showed significant the count of total coliform and total bacterial count. Moreover, total coliform count is also positive associated with total bacterial count of the CPH treated meat during 15 days’ storage. Total bacterial count as no association with color value of the meat like L, a and b value. Color value of the meat showed significant association with a & b value during the entire study duration and a value also showed the increased trend with the enhancement of b value of treated meat.

**Table 10:** Correlation study for CPH treated beef nuggets among different parameters.

	TPC	DPPH	TVBN	POV	TBARS	pH	Coliform	TAB	L	a	b
TPC	1										
DPPH	0.73892	1									
TVBN	− 0.49173	− 0.90853	1								
POV	− 0.47019	− 0.90364	0.970214	1							
TBARS	− 0.6828	− 0.96862	0.909496	0.908614	1						
pH	− 0.59344	− 0.83715	0.877013	0.796139	0.806188	1					
Coliform	− 0.76498	− 0.97218	0.866643	0.872296	0.949872	0.823002	1				
TAB	− 0.87701	− 0.94473	0.797051	0.772508	0.934322	0.781914	0.935578	1			
L	0.067774	0.621435	− 0.84055	− 0.86081	− 0.67481	− 0.57837	− 0.59459	− 0.43504	1		
a	0.281671	0.722301	− 0.8983	− 0.91552	− 0.74766	− 0.71206	− 0.72127	− 0.56492	0.942769	1	
b	0.24558	0.715502	− 0.9258	− 0.90052	− 0.76067	− 0.77703	− 0.69488	− 0.57285	0.932685	0.960029	1

**Table 11:** Stepwise regression for CPH treated beef nuggets among different parameters.

Intercept	Coefficients	Standard error	t stat	p-value	Lower 95%	Upper 95%	Lower 95.0%	Upper 95.0%
	0.686201	0.195198	3.515418	0.003429	0.267544	1.104859	0.267544	1.104859
TPC	0.002076	0.000516	4.024818	0.001254	0.00097	0.003182	0.00097	0.003182
DPPH	− 0.00438	0.001236	− 3.54416	0.003238	− 0.00703	− 0.00173	− 0.00703	− 0.00173
TVBN	− 0.00538	0.001922	− 2.80076	0.014159	− 0.0095	− 0.00126	− 0.0095	− 0.00126
TAB	0.026322	0.005136	5.12484	0.000154	0.015306	0.037338	0.015306	0.037338
b	− 0.0259	0.007626	− 3.39657	0.004342	− 0.04226	− 0.00955	− 0.04226	− 0.00955

## Conclusion

The current research draws attention towards the fact that the use of different concentrations of the peptide powder can significantly affect the oxidative stability and functional properties of meat (beef) because of their well-known nutritional and health benefits. In this study, a weighed concentration of the dried milk casein protein hydrolysate (CPH) powder was added to the raw beef nuggets in order to check their established effects on the oxidative stability as well as the functional capability of the minced beef during refrigeration storage. The CPH powder was added in the meat nuggets at different concentrations to check the effect of the peptide powder on oxidative stability and on the functional properties of ground beef made into nuggets at refrigeration temperature. The results acquired suggest that these anti-oxidant rich or biologically active compounds can effectively retard and or suppress the lipid-oxidation during the storage period. These casein protein hydrolysates exhibit potent anti-oxidant and anti-microbial properties. Results suggested that the buffalo milk casein could be used as a natural source of food protein to produce hydrolysates with higher anti-oxidant and anti-microbial activities. It also encourages the use of milk caseins and derived peptides for direct human consumption and as ample ingredients in reformulated and functional meat and meat products for enhancing their functionalities and shelf-life. Concisely, it can be concluded that the addition of bioactive peptides (BAP’s) in beef nuggets have proved to be efficacious in improving the quality of meat. Its addition in meat or meat based products can help in curbing the deleterious quality effects along with storage of these products or the meat itself and hence can promote health which is the sole purpose of recent therapeutic food development researches and are indeed the need of hour.

## Data Availability

The data generated for this study available in the manuscript.
